# Supplementary material for: Cysteine synthases CYSL-1 and CYSL-2 mediate C. elegans heritable adaptation to P. vranovensis infection
Source: Nat Commun. 2020 Apr 8;11:1741. doi: 10.1038/s41467-020-15555-8 (PMC7142082; doi:10.1038/s41467-020-15555-8)
Supplement: Supplementary file 1 — Supplementary Information [file 41467_2020_15555_MOESM1_ESM.pdf]

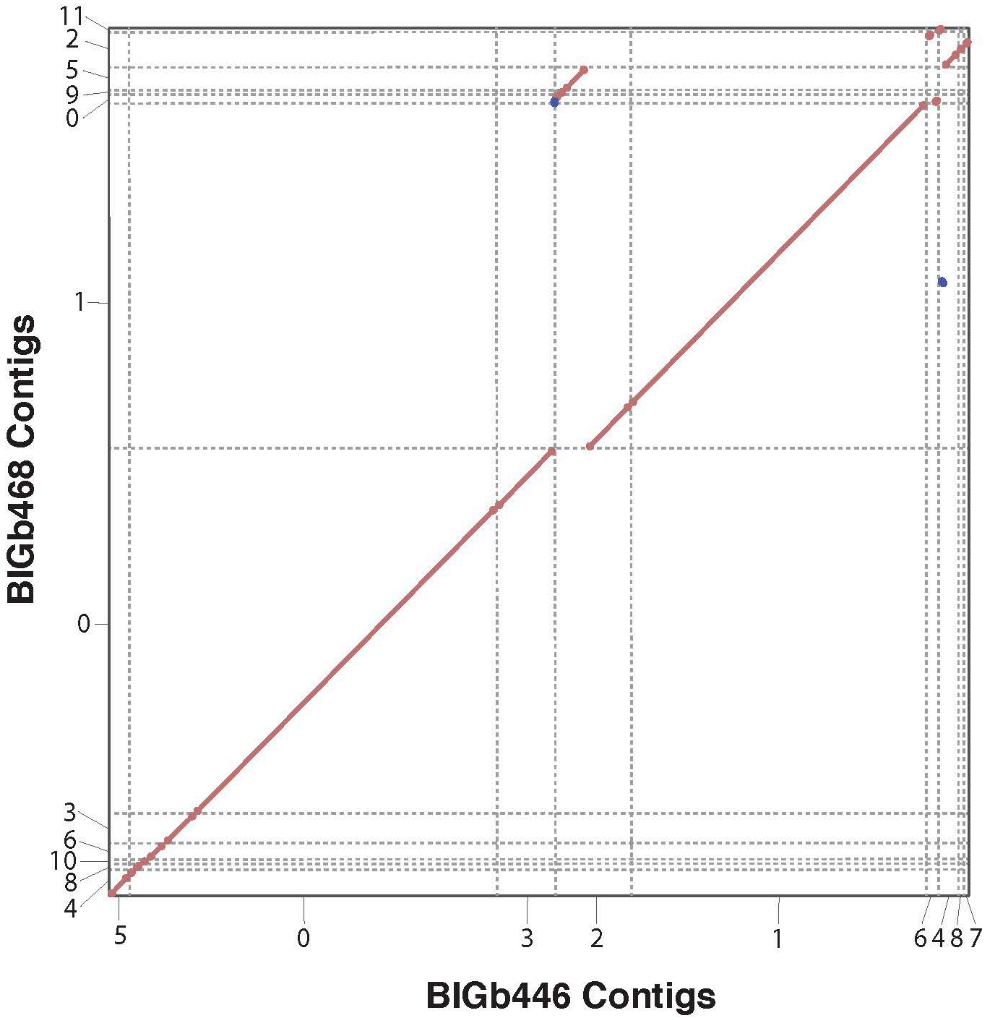

Supplementary Figure 1. BIGb446 and BIGb468 are isolates of a single species. MUMmer plot of the alignments of contigs of BIGb446 and BIGb468 assembled genomes.

a

Fertilization	Maternal Diet	Paternal Diet	Alive	Dead
Self-fertilized on carbenicillin plates	HB101	HB101	2	119
Self-fertilized on carbenicillin plates	BIGb446	BIGb446	1	91
Mated on carbenicillin plates	HB101	BIGb446	1	137

b

Parental Diet	Alive	Dead
HB101	3	97
BIGb446	32	68

c

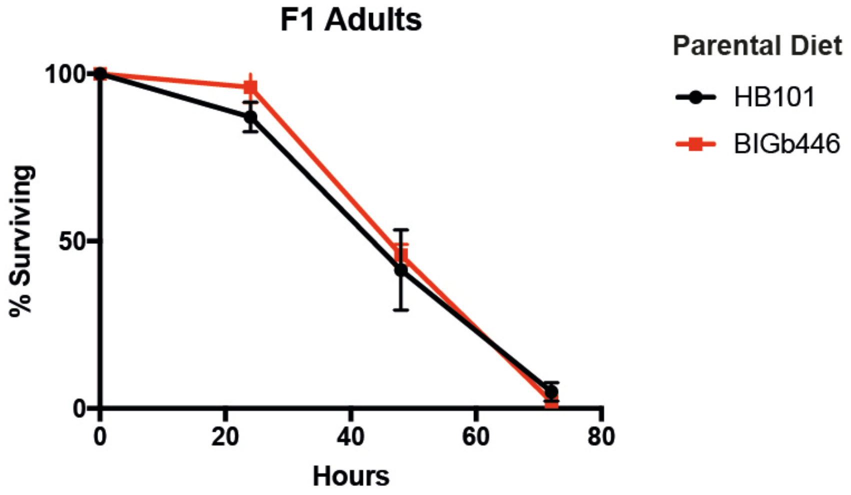

Supplementary Figure 2. Eliminating infection by *P. vranovensis* results in a loss of adaptation. (a) Infected and uninfected male and hermaphrodite wild-type adults were placed onto plates containing carbenicillin for 24 hours. Embryos were transferred to fresh plates seeded with *P. vranovensis* BIGb446 and were assayed for survival after 24 hours. (b) 3-fold stage embryos from parents fed *E. coli* HB101 or *P. vranovensis* BIGb446 for 24 hours were transferred to fresh plates seeded with *P. vranovensis* BIGb446. Survival was assayed after

24 hours. (c) Survival of F1 adults from parents fed a control diet of *E. coli* HB101 or exposed to *P. vranovensis* BIGb446 for 24 hours. F1 larvae developed on plates seeded with *E. coli* HB101. Data presented as mean values +/- s.d. n = 3 experiments of >100 animals. Source data are provided as a Source Data file.

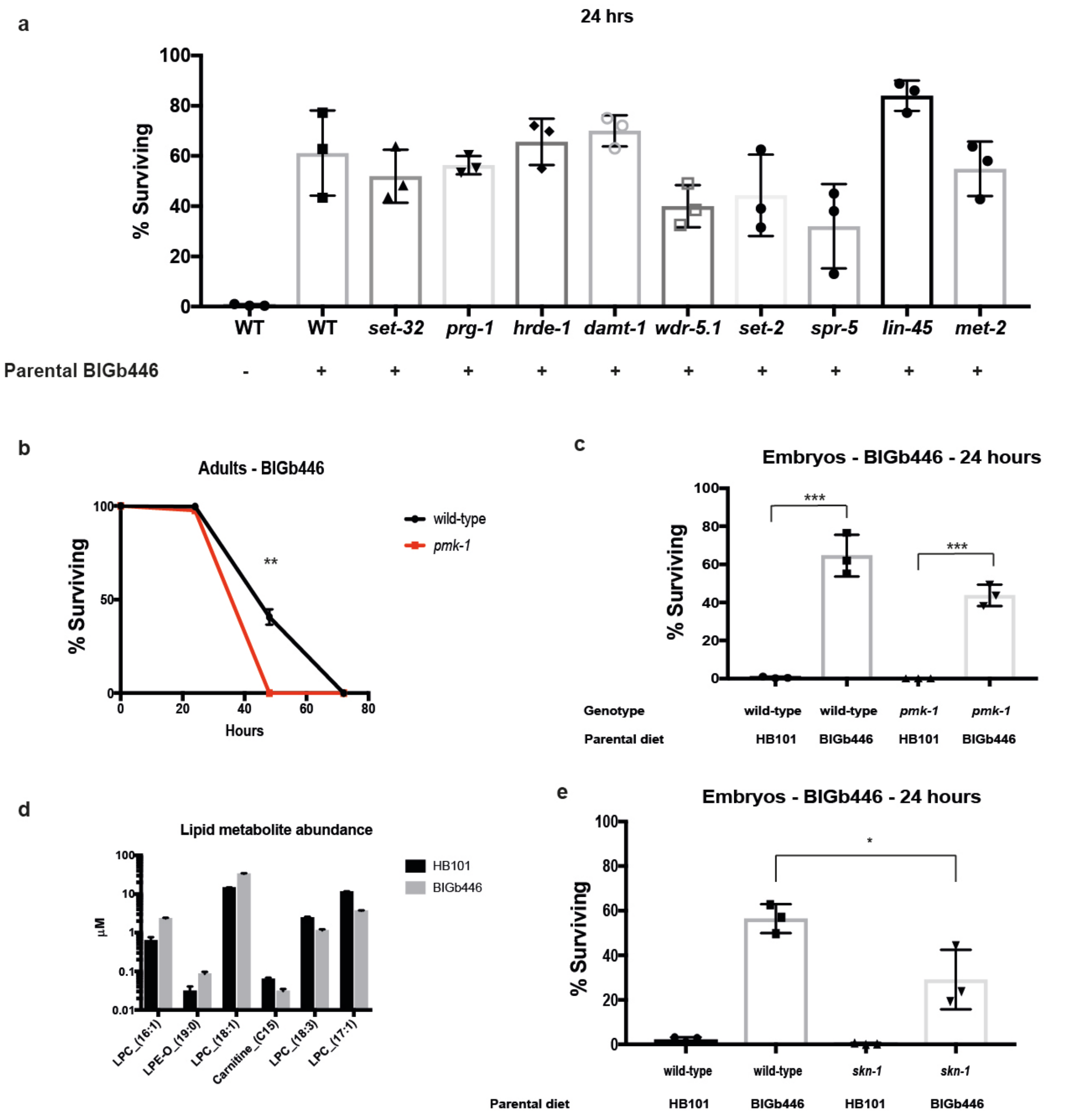

Supplementary Figure 3. Adaptation to *P. vranovensis* does not require factors previously reported to be required for multigenerational effects in *C. elegans*. (a) Percent of wild-type, *set-32(ok1457), prg-1(n4357), hrde-1(tm1200), damt-1(gk961032), wdr-5.1(ok1417), set-2(ok952), spr-5(by134), lin-45(n2018),* and *met-2(n4256)* mutants surviving on plates seeded with bacterial isolates BIGb446 after 24 hrs. Data presented as mean values +/- s.d. n = 3 experiments of >100 animals. (b) Percent of wild-type and *pmk-1(km25)* mutant adults surviving on NGM plates seeded with *P. vranovenis* BIGb446. Data presented as mean

values +/- s.d. n = 3 replicates of 100 animals. (c) Percent of wild-type and *pmk-1(km25)* mutants surviving on plates seeded with bacterial isolate BIGb446 after 24 hrs. Data presented as mean values +/- s.d. n = 3 experiments of >100 animals. *** = $p < 0.001$, **** $p < 0.0001$ (d) μM abundance of lipid metabolites exhibiting a greater than 2-fold change in abundance in embryos from parents fed *P. vranovensis* BIGb446 when compared to embryos from parents fed *E. coli* HB101. Data presented as mean values +/- s.d. n = 3 replicates. (e) Percent of wild-type and *skn-1(zj15)* mutants surviving on plates seeded with *P. vranovensis* BIGb446 after 24 hrs. Data presented as mean values +/- s.d. n = 3 experiments of >100 animals. Source data are provided as a Source Data file. * = $p < 0.05$, ** = $p < 0.01$, *** = $p < 0.001$. See statistics and reproducibility section for statistical tests run.

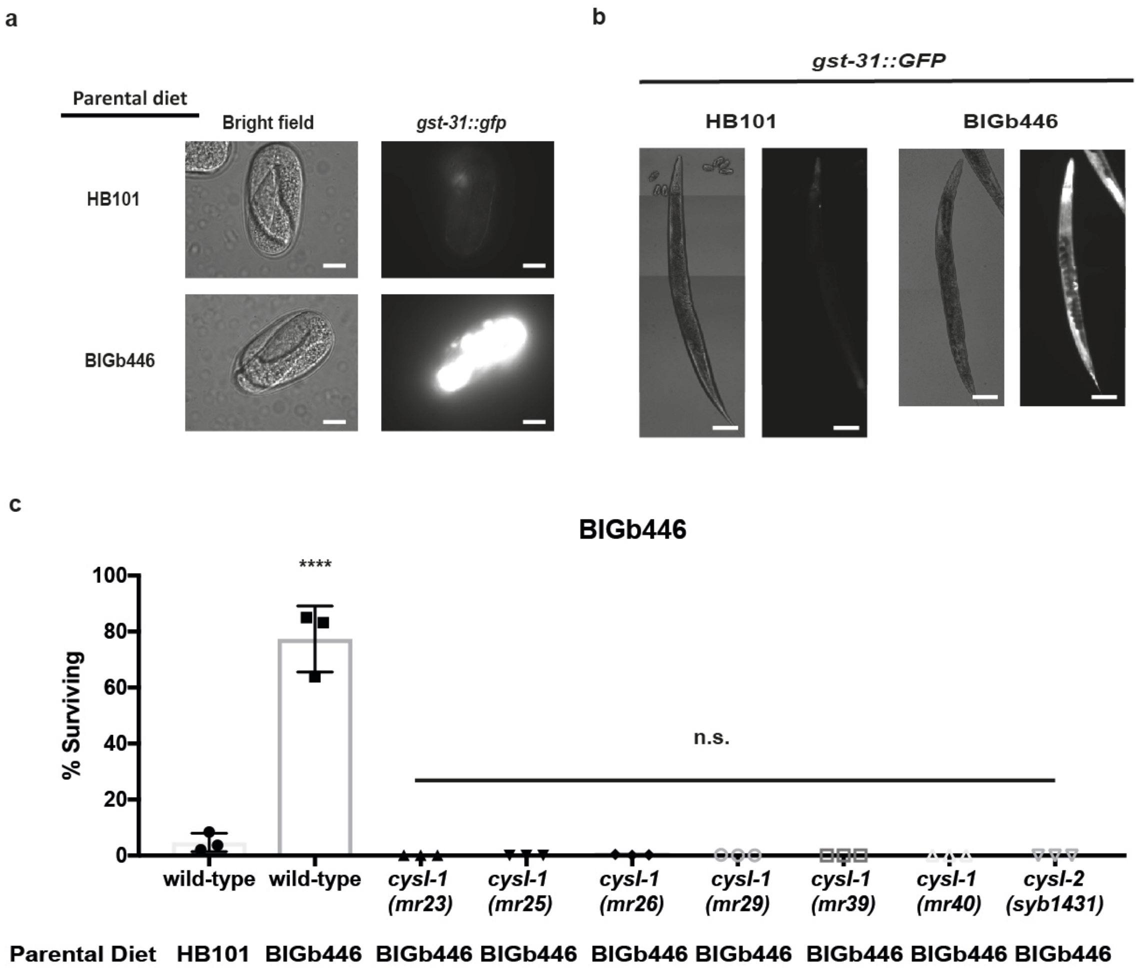

Supplementary Figure 4. Parental exposure to *P. vranovensis* both alters progeny gene expression and promotes progeny adaptation to *P. vranovensis* via a mechanism that requires CYSL-1 and CYSL-2. (a) Representative images of *gst-31::GFP* in 3-fold stage embryos from parents either fed *E. coli* HB101 or exposed to *P. vranovensis* BIGb446. Scale bars 10 µm. (b) Representative images of *gst-31::GFP* in adult animals fed E. coli HB101 or exposed to *P. vranovensis* BIGb446. Scale bars 100 µm. (c) Percent of wild-type and *cysl-1(mr23, mr25, mr26, mr29, mr39, mr40)* and *cysl-2(syb1431)* mutants surviving on plates seeded with bacterial isolates BIGb446 after 24 hrs. Data presented as mean values +/- s.d. n = 3 experiments of >100 animals. **** $p < 0.0001$. Source data are provided as a Source Data file.

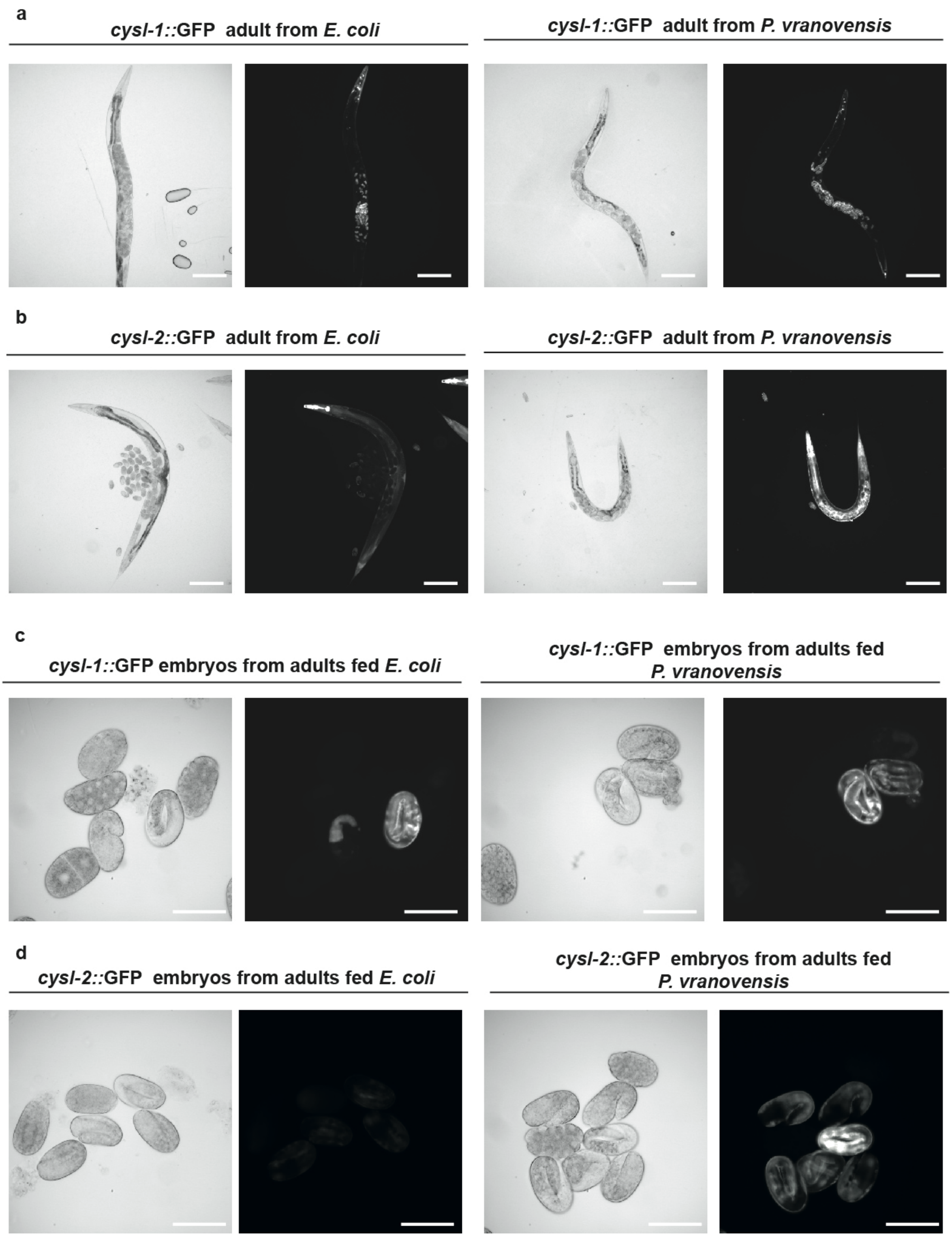

Supplementary Figure 5. *P. vranovensis* infection promotes *cysl-2::GFP* expression in adults and F1 embryos. (a) Representative images of *cysl-1::GFP* in adult animals fed *E. coli* HB101 or exposed to *P. vranovensis* BIGb446. Scale bars 200 µm. (b) Representative images of *cysl-2::GFP* in adult animals fed *E. coli* HB101 or exposed to *P. vranovensis* BIGb446.

Scale bars 200 µm. (c) Representative images of *cysl-1::GFP* in F1 embryos from parents fed *E. coli* HB101 or exposed to *P. vranovensis* BIGb446. Scale bars 50 µm. (d) Representative images of *cysl-2::GFP* in F1 embryos from parents fed *E. coli* HB101 or exposed to *P. vranovensis* BIGb446. Scale bars 50 µm. All experiments repeated three times with similar results.

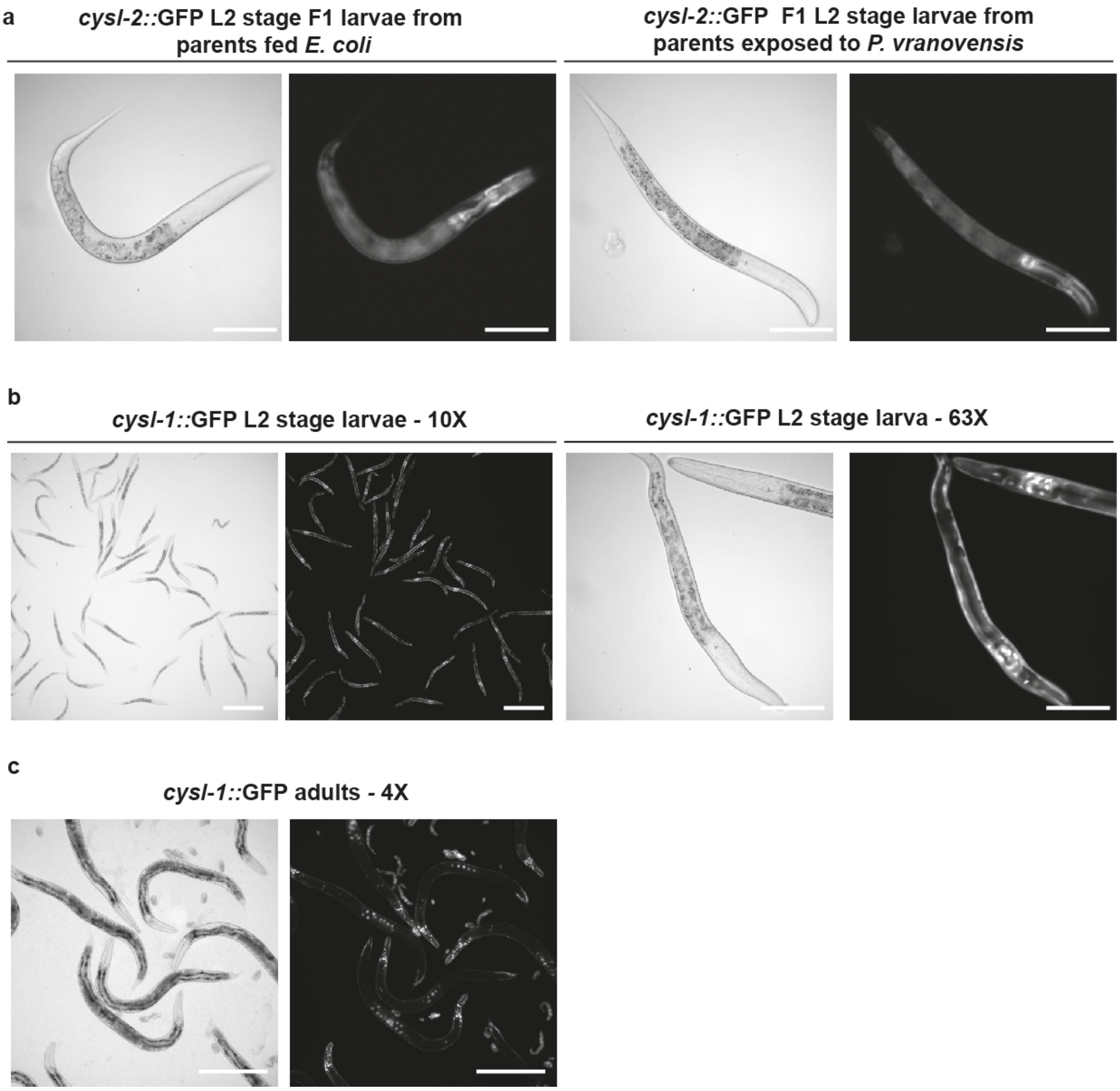

Supplementary Figure 6. *P. vranovensis* infection alters gene expression in *C. elegans*. (a) Representative images of *cysl-2*::GFP in F1 offspring growing on *E. coli* HB101. Parents of F1 offspring fed either *E. coli* HB101 or exposed to *P. vranovensis* BIGb446. Scale bars 50 μm. (b) Representative 10X and 63X images of L2-stage animals expressing *cysl-1::GFP*. Animals fed *E. coli* HB101. Scale bars 200 μm for 10X and 50 μm for 63X. (c) Representative 4X image of adult animals expressing *cysl-1::GFP*. Animals were fed *E. coli* HB101. Scale bars 500 μm. All experiments repeated three times with similar results.
