# Supplementary material for: Cysteine synthases CYSL-1 and CYSL-2 mediate C. elegans heritable adaptation to P. vranovensis infection
Source: Nat Commun. 2020 Apr 8;11:1741. doi: 10.1038/s41467-020-15555-8 (PMC7142082; doi:10.1038/s41467-020-15555-8)
Supplement: Supplementary file 3 — Description of Additional Supplementary Files [file 41467_2020_15555_MOESM3_ESM.docx]

Supplementary Data 1. Genome assembly of *P. vranovensis* BIGb446. FASTA format file of *P. vranovensis* BIGb446 genomic DNA.

Supplementary Data 2. Genome assembly of *P. vranovensis* BIGb468. FASTA format file of *P. vranovensis* BIGb468 genomic DNA.

Supplementary Data 3. Comparison of genomic DNA sequences of BIGb446 and BIGb468 to *P. vranovensis.*

Supplementary Data 4. Profile of lipid metabolite abundances in wild-type embryos from parents fed *E. coli* HB101 or *P. vranovensis* BIGb446. P-values calculated using two-tail t-test with Bonferonni correction.

Supplementary Data 5. DEseq2 and TPM analysis of mRNA expression in wild-type embryos from parents fed *E. coli* HB101 or *P. vranovensis* BIGb446. Differentially expressed genes were identified with DESeq2, this fits a generalized linear model using the negative binomial distribution and preforms a Wald test to calculate P-values, these are subsequently corrected for multiple tests with the Benjamini-Hochberg procedure.

Supplementary Data 6. DEseq2 analysis of mRNA expression in wild-type embryos from parents fed *E. coli* HB101, *P. aeruginosa* PA14, or *P. luminescens* Hb. Differentially expressed genes were identified with DESeq2, this fits a generalized linear model using the negative binomial distribution and preforms a Wald test to calculate P-values, these are subsequently corrected for multiple tests with the Benjamini-Hochberg procedure

Supplementary Data 7. DEseq2 of mRNA expression in wild-type and *wdr-23* mutant embryos from parents fed *E. coli* HB101. Differentially expressed genes were identified with DESeq2, this fits a generalized linear model using the negative binomial distribution and preforms a Wald test to calculate P-values, these are subsequently corrected for multiple tests with the Benjamini-Hochberg procedure

Source Data. Statistics Source Data as Excel table.
