# Supplementary material for: Cysteine synthases CYSL-1 and CYSL-2 mediate C. elegans heritable adaptation to P. vranovensis infection
Source: Nat Commun. 2020 Apr 8;11:1741. doi: 10.1038/s41467-020-15555-8 (PMC7142082; doi:10.1038/s41467-020-15555-8)
Supplement: Supplementary file 11 — Reporting Summary [file 41467_2020_15555_MOESM11_ESM.pdf]

nature research

Corresponding author(s): Nicholas Burton

Last updated by author(s): Mar 10, 2020

Reporting Summary

Nature Research wishes to improve the reproducibility of the work that we publish. This form provides structure for consistency and transparency in reporting. For further information on Nature Research policies, see Authors & Referees and the Editorial Policy Checklist .

Statistics

For all statistical analyses, confirm that the following items are present in the figure legend, table legend, main text, or Methods section.

n/a	Confirmed	
☐	☒	The exact sample size (*n*) for each experimental group/condition, given as a discrete number and unit of measurement
☐	☒	A statement on whether measurements were taken from distinct samples or whether the same sample was measured repeatedly
☐	☒	The statistical test(s) used AND whether they are one- or two-sided<br>*Only common tests should be described solely by name; describe more complex techniques in the Methods section.*
☒	☐	A description of all covariates tested
☐	☒	A description of any assumptions or corrections, such as tests of normality and adjustment for multiple comparisons
☐	☒	A full description of the statistical parameters including central tendency (e.g. means) or other basic estimates (e.g. regression coefficient) AND variation (e.g. standard deviation) or associated estimates of uncertainty (e.g. confidence intervals)
☐	☒	For null hypothesis testing, the test statistic (e.g. *F*, *t*, *r*) with confidence intervals, effect sizes, degrees of freedom and *P* value noted<br>*Give P values as exact values whenever suitable.*
☒	☐	For Bayesian analysis, information on the choice of priors and Markov chain Monte Carlo settings
☒	☐	For hierarchical and complex designs, identification of the appropriate level for tests and full reporting of outcomes
☒	☐	Estimates of effect sizes (e.g. Cohen's *d*, Pearson's *r*), indicating how they were calculated

Our web collection on statistics for biologists contains articles on many of the points above.

Software and code

Policy information about availability of computer code

Data collection	No data collection software were used in this manuscript.
Data analysis	Bioconductor DESeq2 R package v3.5.1 Love et al., 2014 https:.//anaconda.org<br>HISAT v2.1.0 Kim et al., 2015 https:.//anaconda.org<br>samtools v1.9 Li et al., 2009 https:.//anaconda.org<br>FeatureCounts v1.6.3 Liao et al., 2014 https:.//anaconda.org<br>cutadapt v1.18 Marcel Martic 2011 https:.//anaconda.org<br>MUMmer v3.0 and MUMmer-plot Kurtz et al., 2004 https://mummer4.github.io/manual/manual.html<br>NUCmer v3.23 (from MUMmer package) Kurtz et al., 2004 https://mummer4.github.io/manual/manual.html<br>dnadiff v3.23 (from MUMmer package) Kurtz et al., 2004 https://mummer4.github.io/manual/manual.html<br>SMRT Link v8.0 https://www.pacb.com/wp-content/uploads/SMRT-Link-User-Guide-v8.0.pdf<br><br>A snakemake workflow (CITE snakemake (Köster and Rahmann, 2018)) was created for the RNA-seq analysis and can it be found at https://github.com/cristianriccio/celegans-pathogen-adaptation.

For manuscripts utilizing custom algorithms or software that are central to the research but not yet described in published literature, software must be made available to editors/reviewers. We strongly encourage code deposition in a community repository (e.g. GitHub). See the Nature Research guidelines for submitting code & software for further information.

Data

Policy information about availability of data

All manuscripts must include a data availability statement. This statement should provide the following information, where applicable:

- Accession codes, unique identifiers, or web links for publicly available datasets
- A list of figures that have associated raw data
- A description of any restrictions on data availability

Genomic DNA and RNA-seq data that support the findings of this study have been deposited at the European Nucleotide Archive under the accession code PRJEB32993. The raw data related to metabolomics is available on Metabolomics Workbench using the DataTrack ID 1891.

Field-specific reporting

Please select the one below that is the best fit for your research. If you are not sure, read the appropriate sections before making your selection.

[x] Life sciences [ ] Behavioural & social sciences [ ] Ecological, evolutionary & environmental sciences

For a reference copy of the document with all sections, see nature.com/documents/nr-reporting-summary-flat.pdf

Life sciences study design

All studies must disclose on these points even when the disclosure is negative.

Sample size	Sample sizes were chosen to compare to the literature in the field -- Burton et al., 2017, Burton et al., 2018, and Moore et al., 2019.
Data exclusions	No data was excluded.
Replication	All experiments were repeated at least 3 times and replication was successful on all occasions.
Randomization	Embryos from all animals/genotypes were collected and randomly allocated to different experimental plates.
Blinding	Experimenters were not blinded during this study as all animals were quantified and all quantifications were fully reported without any exclusions.

Reporting for specific materials, systems and methods

We require information from authors about some types of materials, experimental systems and methods used in many studies. Here, indicate whether each material, system or method listed is relevant to your study. If you are not sure if a list item applies to your research, read the appropriate section before selecting a response.

Materials & experimental systems

n/a	Involved in the study	
[x]	[ ]	Antibodies
[x]	[ ]	Eukaryotic cell lines
[x]	[ ]	Palaeontology
[ ]	[x]	Animals and other organisms
[x]	[ ]	Human research participants
[x]	[ ]	Clinical data

Methods

n/a	Involved in the study	
[x]	[ ]	ChIP-seq
[x]	[ ]	Flow cytometry
[x]	[ ]	MRI-based neuroimaging

Animals and other organisms

Policy information about studies involving animals; ARRIVE guidelines recommended for reporting animal research

Laboratory animals	C. elegans (nematode) all larval stages and adult hermaphrodites of the N2 genetic background unless specific mutation noted in methods; P. vranovensis, P. luminescens, P. aeruginosa (bacteria)
Wild animals	This study did not involve wild animals.
Field-collected samples	This study did not involve animals collected from the field.
Ethics oversight	No ethical approval was required as this study did not use any vertebrates or controlled animal species.

Note that full information on the approval of the study protocol must also be provided in the manuscript.
